# Supplementary material for: Addressing the Molecular Mechanism of Longitudinal Lamin Assembly Using Chimeric Fusions
Source: Cells. 2020 Jul 7;9(7):1633. doi: 10.3390/cells9071633 (PMC7407374; doi:10.3390/cells9071633)
Supplement: Supplementary file 1 [file cells-09-01633-s001.pdf]

Addressing the molecular mechanism of longitudinal lamin assembly using chimeric fusions

Giel Stalmans [1,†], Anastasia V. Lilina [1,†], Pieter-Jan Vermeire [1], Jan Fiala [2,3], Petr Novák [2,3] and Sergei V. Strelkov [1,*]

[1] Laboratory for Biocrystallography, KU Leuven, 3000 Leuven, Belgium; giel.stalmans@kuleuven.be (G.S.); anastasia.lilina@kuleuven.be (A.V.L.); pieterjan.vermeire@kuleuven.be (P.-J.V.)
[2] Department of Biochemistry, Charles University, 128 00 Prague, Czech Republic; jan.fiala@biomed.cas.cz (J.F.); pnovak@biomed.cas.cz (P.N.)
[3] Institute of Microbiology of the Czech Academy of Sciences, 142 20 Prague, Czech Republic; jan.fiala@biomed.cas.cz, pnovak@biomed.cas.cz
* Correspondence: sergei.strelkov@kuleuven.be; Tel.: +32-1633-0845
† These authors have contributed equally to this work.

Supplementary material

Supplementary Table 1. DNA primers used.

Primers for Quick-Change™ Site-Directed Mutagenesis			
		**Forward primers**	**Reverse primers**
**Gp7$_{F40C}$-LA 327-403**	*Gp7$_{F40C}$*	CGGTTCTTGCGTGTCCGAGTAC AATGATTTAAC	TCGGACACGCAAGAACCGTAGT TCACTCTGAG
**Primers for sequence and ligation-independent cloning**			
		**Forward primers**	**Reverse primers**
**LamA 22-70-Eb1**	*LA 22-70*	GCGAACAGATTGGTGGTTCGCC CACCCGCATCACC	TCCGAAGTAGAAATCGACCACC TCTTCAGACTCGG[1]
	Eb1	GATTTCTACTTCGGAAAGCTAC[2]	TTGTTAGCAGAAGCTTATTATTC AT[3]
**LamA 17-70-Eb1**	*LA 17-70*	GCGAACAGATTGGTGGTAGCTC CACTCCGCTGTCGC	[1]
	Eb1	[2]	[3]
**LamA 1-70-Eb1**	*LamA 1-70*	GCGAACAGATTGGTGGTATGGA GACCCCGTCCCAGC	[1]
	Eb1	[2]	[3]
**Gp7$_{F40C}$-LA 327-403**	*LA 327-403*	GATTTAACAAAATCTCTGGCCC GTGAGCGGGAC	TTGTTAGCAGAAGCTTATTAGG AAGCACGGCCACGGCT
	Gp7$_{F40C}$	GCGAACAGATTGGTGGTGGC	AGATTTTGTTAAATCATTGTACT CG

Supplementary Table 2. X-ray diffraction and refinement statistics.

	**GP7$_{F40C}$-LA 327-403**		**LA 17-70-Eb1**	**LA 1-70-Eb1**
**Sequence features**				
No. of residues	127[49]		91 [37]	107 [37]
Molecular weight (kDa)	14.872		10.597	12.326
No. of Cys residues	1 [1]		1 [1]	1 [1]
No. of disulphides per dimer	1 [1]		0 [0]	0 [0]
No. of Met residues	5 [1]		0 [0]	1 [0]
**Diffraction dataset**	**Native**	**Anomalous**		
Number of crystals used	1		1	1
Number of datasets collected	1	3	1	1
Space group	$P\ 6_1\ 2\ 2$		$P\ 2_1\ 2_1\ 2_1$	$I\ 4_1\ 2\ 2$
Unit cell dimensions:				

a, b, c (Å)	117.25, 117.25, 93.16		27.37, 105.24, 119.77	108.85,108.85,130,47
α, β, γ (°)	90, 90, 120		90, 90, 90	90, 90, 90
Resolution range (Å)	44.6-2.9 (3.0-2.9)	42.7-3.2 (3.3-3.2)	52.05 - 1.83 (1.86 - 1.83)	76.9-2.8 (2.9-2.8)
<I/σ>	20.27 (0.99)	60.19 (5.95)	9.73 (1.08)	11.52 (1.85)
$CC_{½}$ (%)	0.999 (0.934)	1 (0.998)	0.999 (0.540)	0.999 (0.865)
Completeness (%)	98.28 (98.24)	98.06 (94.26)	100.00 (98.54)	99.44 (98.73)
No. of unique reflections	8792 (847)	11862 (1181)	31710 (1487)	9656 (939)
Redundancy	19.2 (20.1)	122.2 (84.3)	10.2 (7.6)	26.4 (27.1)
Wilson B-factor ($Å^2$)	110.6	129.5	22.2	76.0
Solvent content (%)	80.2		39.5	68.6
**Refinement**				
R_{work} (%)	25.33		22.50	23.75
R_{free} (%)	30.42		25.32	28.13
No. of protein chains per a.s.u.	1		4	2
No. of non-hydrogen atoms:				
protein	823		2842	1382
ligands/ions	19		0	0
water molecules	2		86	22
R.m.s. deviations:				
bonds (Å)	0.017		0.014	0.018
angles (°)	2.17		1.78	2.20
Ramachandran plot: favoured/allowed/outlier (%)	98/2.0/0		99.7/0.3/0	98.8/1.2/0
Sidechain rotamer outliers (%)	9.4		4.7	5.9
Clash score	2.43		4.24	11.84

Residue counts in square brackets refer to the capping motif. Statistics in round brackets are for the highest resolution bin.

Supplementary Table 3. Chemical cross-links obtained for the complexes of N- and C-terminal fragments.

#	Protein 1	Protein 2	Pos 1	Pos 2	Crosslinker	Linkage	Distance (Å)
1	LA 22-70-Eb1	LA 22-70-Eb1	K76	S66	DSBU	monomer/dimer	15.1
2	LA 22-70-Eb1	LA 22-70-Eb1	K76	T64	DSBU	monomer/dimer	18.2
3	LA 17-70-Eb1	LA 17-70-Eb1	S17	K32	DSPU	monomer/dimer	5.3
4	LA 22-70-Eb1	LA 22-70-Eb1	S22	Y45	DSBU	monomer/dimer	24.4
5	LA 22-70-Eb1	LA 22-70-Eb1	S22/T24	K32	DSBU	monomer/dimer	5.3
6	LA 22-70-Eb1	LA 22-70-Eb1	T27	K32	DSBU & DSG	monomer/dimer	5.3
7	LA 22-70-Eb1	LA 22-70-Eb1	Y45	K32	DSBU & DSG	monomer/dimer	19.7
8	Gp7$_{F40C}$-LA 327-403	Gp7$_{F40C}$-LA 327-403	G277	K291	DSG	monomer/dimer	21.9
9	Gp7$_{F40C}$-LA 327-403	Gp7$_{F40C}$-LA 327-403	G277	K325	DSBU & DSPU	monomer/dimer	18.1
10	Gp7$_{F40C}$-LA 327-403	Gp7$_{F40C}$-LA 327-403	K281	K325	DSBU & DSPU	monomer/dimer	23.6
11	Gp7$_{F40C}$-LA 327-403	Gp7$_{F40C}$-LA 327-403	K281	S300	DSG	monomer/dimer	23.0

12	$Gp7_{F40C}$-LA 327-403	$Gp7_{F40C}$-LA 327-403	K325	S318	DSBU & DSG	monomer/dimer	17.1
13	$Gp7_{F40C}$-LA 327-403	$Gp7_{F40C}$-LA 327-403	K341	G277	DSBU & DSPU	monomer/dimer	37.5
14	$Gp7_{F40C}$-LA 327-403	$Gp7_{F40C}$-LA 327-403	S326	T324	DSG	monomer/dimer	5.6
15	$Gp7_{F40C}$-LA 327-403	$Gp7_{F40C}$-LA 327-403	Y313	K281	DSPU	monomer/dimer	11.5
16	$Gp7_{F40C}$-LA 327-403	$Gp7_{F40C}$-LA 327-403	K281	K341	DSBU	dimer	41.3
17	LA 17-70-Eb1	LA 17-70-Eb1	S17	S17	DSPU	dimer	19.8
18	LA 22-70-Eb1	LA 22-70-Eb1	S22	S22	DSBU	dimer	19.8
19	LA 17/22-70-Eb1	LA 17/22-70-Eb1	K32	K32	DSBU & DSG & DSPU	dimer	16.0
20	$Gp7_{F40C}$-LA 327-403	$Gp7_{F40C}$-LA 327-403	K341	K341	DSBU & DSG & DSPU	dimer	11.7
21	$Gp7_{F40C}$-LA 327-403	$Gp7_{F40C}$-LA 327-403	K378	K378	DSBU & DSG	dimer	21.9
22	LA 22-70-Eb1	$Gp7_{F40C}$-LA 327-403	K32	K341	DSBU & DSG	tetramer	8.1
23	LA 17-70-Eb1	$Gp7_{F40C}$-LA 327-403	K32	Y313	DSPU	tetramer	41.9
24	LA 17-70-Eb1	$Gp7_{F40C}$-LA 327-403	S17	G277	DSPU	tetramer	33.1
25	LA 17-70-Eb1	$Gp7_{F40C}$-LA 327-403	S17	K341	DSPU	tetramer	11.4
26	LA 17-70-Eb1	$Gp7_{F40C}$-LA 327-403	S17	Y359	DSPU	tetramer	30.1
27	LA 22-70-Eb1	$Gp7_{F40C}$-LA 327-403	S22	K341	DSBU	tetramer	11.4
28	LA 22-70-Eb1	$Gp7_{F40C}$-LA 327-403	T105	Y376	DSBU	tetramer	18.8
29	LA 22-70-Eb1	$Gp7_{F40C}$-LA 327-403	T27	S318	DSG	tetramer	29.8
30	$Gp7_{F40C}$-LA 327-403	LA 22-70-Eb1	E381/ E383	K76	EDC	tetramer	12.2
31	$Gp7_{F40C}$-LA 327-403	LA 22-70-Eb1	K378	E65	EDC	tetramer	11.7
32	LA 17-70-Eb1	$Gp7_{F40C}$-LA 327-403	T105	Y359	DSPU	/	/
33	LA 22-70-Eb1	LA 22-70-Eb1	K32	K76	DSBU & DSG	/	/
34	LA 22-70-Eb1	LA 22-70-Eb1	K32	T64	DSBU &DSG	/	/
35	LA 22-70-Eb1	LA 22-70-Eb1	S22	K76	DSBU	/	/
36	LA 22-70-Eb1	LA 22-70-Eb1	S22	T64	DSBU	/	/
37	LA 17/22-70-Eb1	$Gp7_{F40C}$-LA 327-403	K32	K366	DSG&DSPU	/	/
38	LA 17/22-70-Eb1	$Gp7_{F40C}$-LA 327-403	K32	K378	DSBU & DSG & DSPU	/	/
39	LA 17-70-Eb1	$Gp7_{F40C}$-LA 327-403	S17	K366	DSPU	/	/
40	LA 17-70-Eb1	$Gp7_{F40C}$-LA 327-403	S17	K378	DSPU	/	/

41	LA 22-70-Eb1	Gp7$_{F40C}$-LA 327-403	S22	K366	DSBU	/	/
42	LA 22-70-Eb1	Gp7$_{F40C}$-LA 327-403	S22	K378	DSBU	/	/
43	LA 22-70-Eb1	Gp7$_{F40C}$-LA 327-403	T105	G277	DSG	/	/
44	Gp7$_{F40C}$-LA 327-403	Gp7$_{F40C}$-LA 327-403	K341	K378	DSBU & DSPU	/	/
45	Gp7$_{F40C}$-LA 327-403	Gp7$_{F40C}$-LA 327-403	K378	S395	DSBU & DSPU	monomer/dimer	/
46	Gp7$_{F40C}$-LA 327-403	Gp7$_{F40C}$-LA 327-403	K378	S392	DSBU & DSPU & DSG	monomer/dimer	/
47	Gp7$_{F40C}$-LA 327-403	Gp7$_{F40C}$-LA 327-403	K378	S390	DSBU	monomer/dimer	/
48	Gp7$_{F40C}$-LA 327-403	Gp7$_{F40C}$-LA 327-403	K366	S395	DSBU	monomer/dimer	/
49	Gp7$_{F40C}$-LA 327-403	Gp7$_{F40C}$-LA 327-403	K366	S392	DSBU & DSPU	monomer/dimer	/
50	Gp7$_{F40C}$-LA 327-403	Gp7$_{F40C}$-LA 327-403	T324	S395	DSBU	/	/
51	Gp7$_{F40C}$-LA 327-403	Gp7$_{F40C}$-LA 327-403	S392/S390	S318	DSG	/	/

All cross-links were obtained in triplicate except for the two EDC cross-links that were technical duplicates. Cross-links that are satisfied in the heterotetramer model (Figure 8) are highlighted in green, red and magenta (EDC cross-links). Green indicates cross-links connecting the chains of the same type (either intrachain cross-links or cross-links occurring within the N- or the C-terminal dimer). Red/magenta indicates the cross-links that interconnect chains of different type (N- *vs* C-terminal). Cross-links in blue are not satisfied in the heterotetramer model but can be explained by further association of two heterotetramers (Supplementary Figure 6). The remaining cross-links (yellow) belong to the flexible tail part that was not present in the model. The maximum allowed distances for each cross-linker were as follows: EDC, 15 Å; DSBU, 40 Å; DSPU, 35 Å; DSG, 30 Å.

Supplementary Table 4. Properties of the final A$_{CN}$ model as analysed using program PISA [1].

Assembly type	Chain 1	Chain 2	Interface area, Å2	ΔiG, kcal/mol	P-value
Intradimer	Gp7$_{F40C}$-LA 327-403 (C∣D)		1686.8	-28.6	0.093
	LA 17-70-Eb1 (A∣B)		880.1	-17.2	0.150
Intratetramer	A	C	1344.9	-16.2	0.318
	B	D	1298.8	-13.5	0.493
	A	D	1094.7	-12.8	0.387
	B	C	1079.2	-14.1	0.306

ΔiG is defined as the change in the solvation energy upon interface formation. This value thus does not include the contribution of hydrogen bonds or salt bridges. The P-value of the interface is defined as the probability to get a lower ΔiG value than measured by chance. For specific hydrophobic interfaces the P-value should be below 0.5.

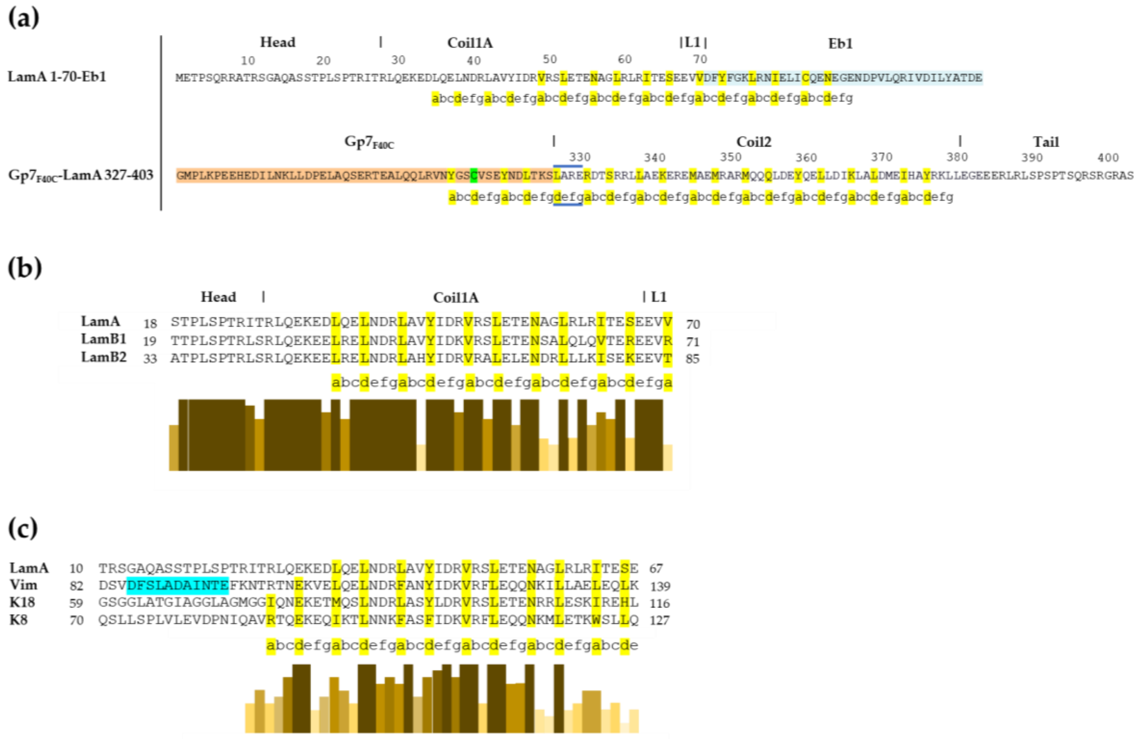

Supplementary Figure 1. Amino-acid sequences. (**a**) The longest N-terminal and the C-terminal LA rod fragment used. Capping motifs Eb1 and Gp7F40C are highlighted in cyan and wheat, respectively. The F40C mutation in Gp7F40C is highlighted in green. CC heptad positions 'a' and 'd' are highlighted in yellow. The stutter region in Gp7F40C-LamA 327-403 is indicated between blue lines. (**b**) Sequence alignment of the proximal part of the head domain and coil1A of human lamins A, B1 and B2. Sequence conservation as determined by the Clustal Omega tool [2] is shown as a bar chart. (**c**) Sequence alignment of the corresponding regions in vimentin (type III IF chain), LA (type V), K18 (type I) and K8 (type II). The predicted 'pre-coil' domain in vimentin is highlighted in cyan.

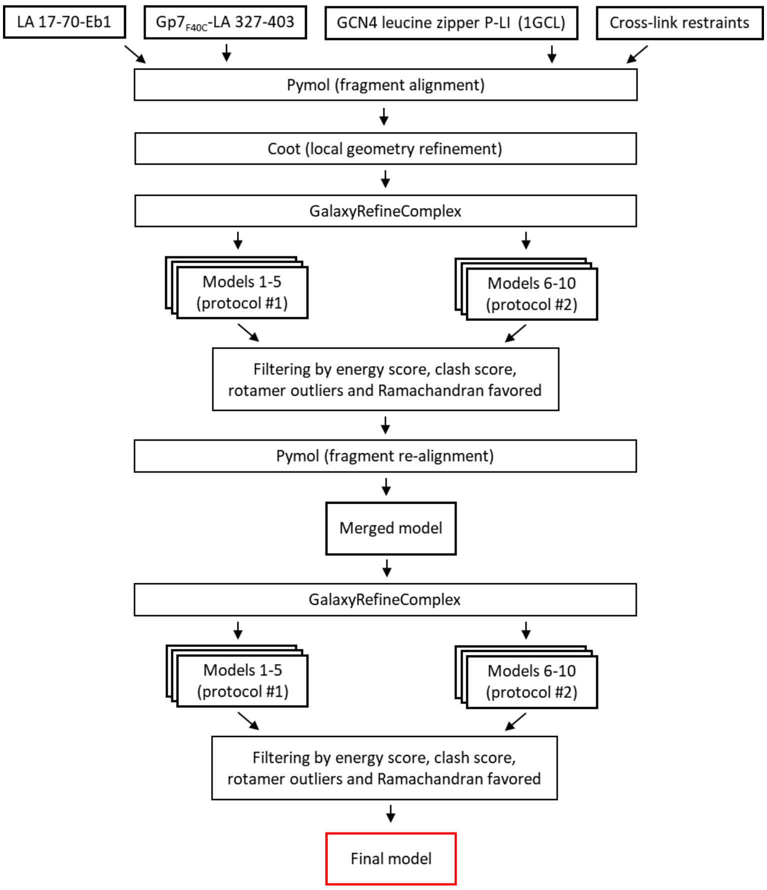

Supplementary Figure 2. Flowchart of molecular modelling and refinement of the heterotetramer. After the initial manual step, the refinement using the GalaxyRefineComplex algorithm [3] yielded two sets of models, each generated by different protocols. Protocol #1 applied only distance restrains and protocol #2 included distance as well as position restrains (for more detail, please refer to Heo et al. [3]). The resulting models from both protocols had limitations. Protocol #1 resulted in misalignment of chains within the tetrameric overlap. Protocol # 2 resulted in a broken disulphide bond in the Gp7$_{F40C}$ cap but maintained a correct alignment of chains A and B. Hence, chains C and D from the best model of protocol #1 and chains A and B from the best model of protocol #2 were merged and re-refined. The latter resulted in the final model (Figure 8).

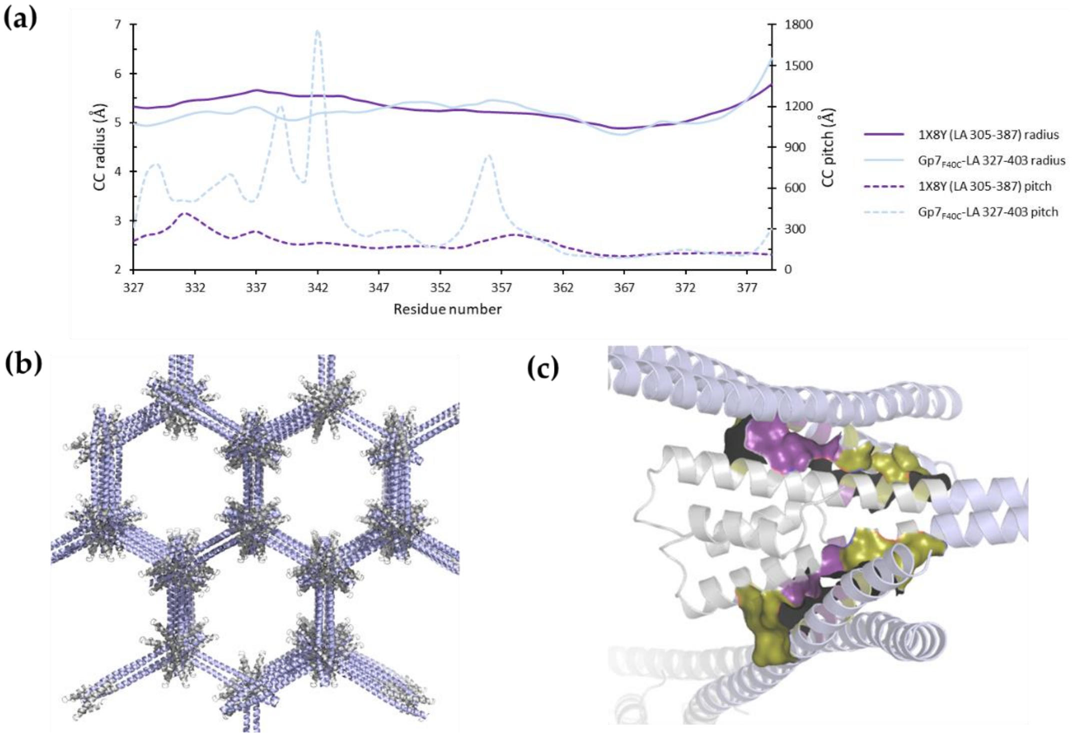

Supplementary Figure 3. Analysis of the Gp7F40C-LA 327-403 structure. (**a**) CC geometry analysis in comparison with the previously determined LA 305-387 fragment (PDB code 1X8Y [4]). The CC radius variation along the length of the two structures as determined using the TWISTER program [5] is given by blue and purple solid lines respectively. The corresponding CC pitch values are plotted as dotted lines. (**b**), (**c**) Crystal arrangement of Gp7F40C-LA 327-403 crystal lattice in two orthogonal views. The LA 327-403 region and Gp7F40C cap are coloured blue and grey, respectively. The Gp7F40C cap is located at the corners of the hexagonal lattice, making key contacts. The hydrophobic core (Val311, Val316, Leu356, Leu363, Tyr359) is coloured purple. The hydrogen bonds (Gln307|Gln360, Gln307|Asp357, Asp370|Asn321) and ionic interactions (Asp370|Lys325, Lys366|Asp322) are coloured olive.

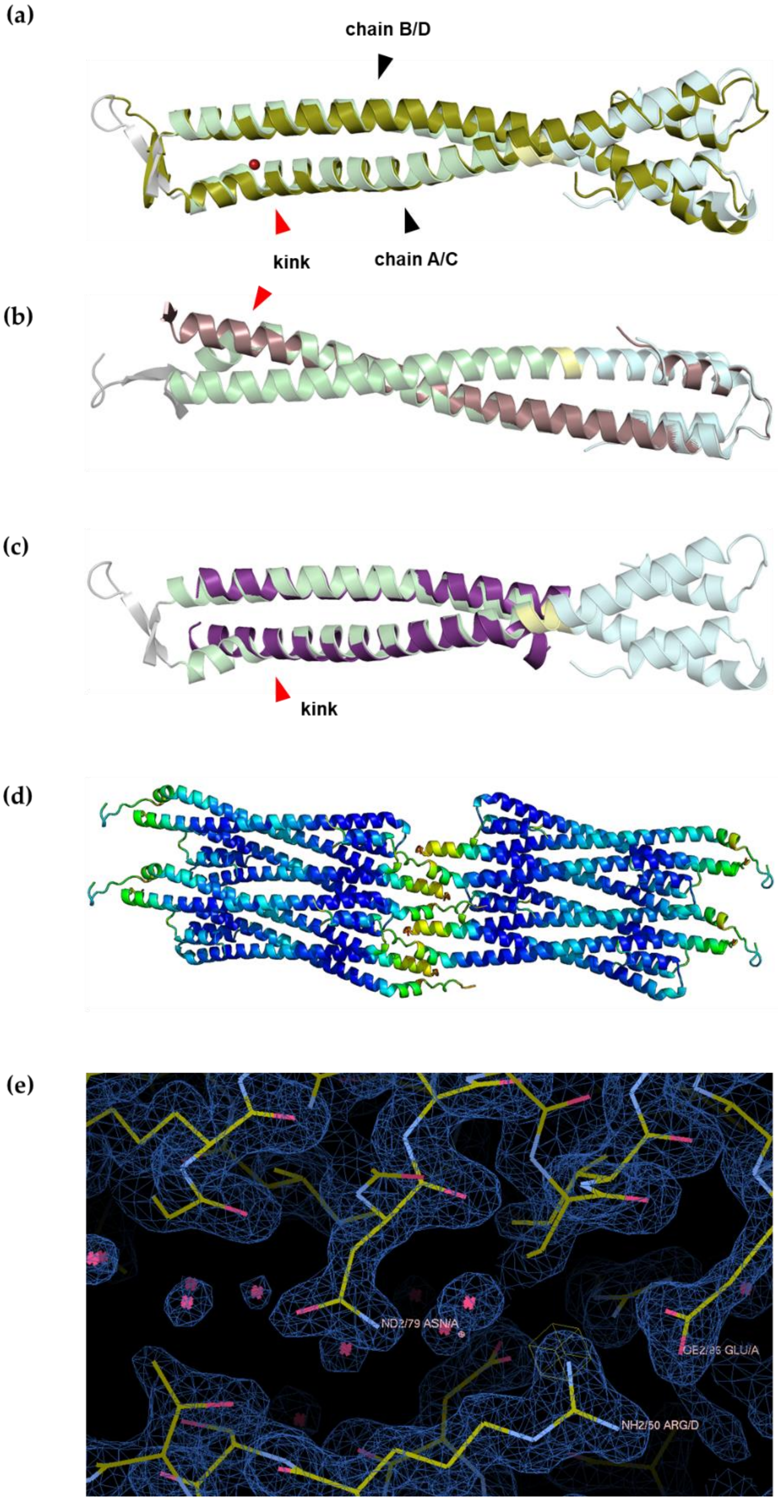

Supplementary Figure 4. Crystal structure of the LA 17-70-Eb1 fusion. (**a**) Superposition of the two dimers AB (grey-green-yellow-cyan) and CD (olive) found in the asymmetric unit. The α-helical kink present at residue Leu35 in chains A and C is indicated. The chains themselves are indicated by black arrows. (**b**) Ribbon diagram of dimer AB, with chain A (with a kink) and chain B (without a kink). Additionally, a copy of chain B (chocolate) has been fitted onto chain A, to demonstrate that the formation of the 'β-lock' is not possible without a kink in one of the α-helices. (**c**) Superposition of the coil1A segments present in the LA 17-70-Eb1 structure (grey-green-yellow-grey) and the recently published LA 1-300 structure (purple, PDB code 6JLB, chains CD [6]). (**d**) Crystal packing arrangement of LA 17-70-Eb1 coloured by B-factors from deep blue (15 Å^2) to orange (80 Å^2). Crystal

contacts with the lowest B-factors are made by the Eb1 cap. **(e)** Example of the 2Fo-Fc electron density map (1.7σ level) for the well-ordered crystal contact produced by the Eb1 cap.

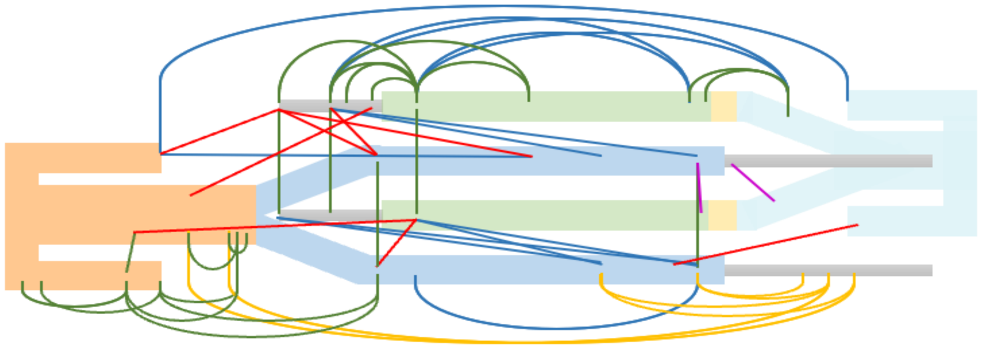

Supplementary Figure 5. Topological scheme of a heterotetramer involving an overlap of partially unzipped N- and C-terminal dimers. All 51 chemical cross-links established for the complex (Supplementary Table 3) are shown as coloured lines. Cross-links that fit the model are coloured green (monomer/dimer) and red (tetramer), cross-links that can only be explained by association of two tetramers (Supplementary Figure 6) are in blue, and cross-links involving the unresolved tail region and not used for modelling are in orange.

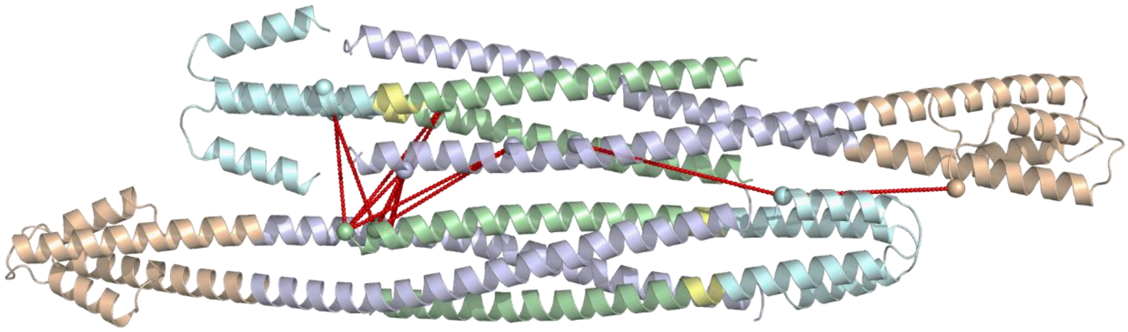

Supplementary Figure 6. Association of two Acn tetramers which satisfies all observed crosslinks (Supplementary Table 3). Only the intertetrameric cross-links are shown (red).

Supplementary Text 1. Detailed information on cross-link identification using MS.

Prior to the liquid chromatography-mass spectrometry (LC-MS) analysis, digestion mixture was reconstituted with 30 µL of 0.1% formic acid. The LC-MS/MS analysis was performed as described in Fiala et al [7]. One µg of cross-linked LA peptides was injected on desalting pre-column (Luna Omega Polar C18 5 µm, 0.3 × 30 mm, Phenomenex, USA) followed by analytical column (Luna Omega Polar C18 3 µm, 0.3 × 150 mm, Phenomenex, USA) heated to 50 °C using Agilent 1290 (Agilent Technologies, USA). The flow rate was 10 µL/min and acetonitrile gradient 5%–35% (*v*/*v*) at 35 min was used. The chromatographic system was directly coupled to solariX XR FT-ICR mass spectrometer (Bruker Daltonics, Germany). Eluted peptides were analysed in positive broadband mode with 1M transient data points over the range 250–2500 m/z. Data-independent acquisition mode was used while ESI-TOF tuning mix (Agilent Technologies, USA) served as a lock mass (m/z 922.0098) and as a centre of isolation window of ± 500 Da for MS/MS. Product ion spectra were acquired at 22.5 eV for DSG and EDC and 17.5 eV for DSBU and DSPU cross-linked peptides.

References:

1. Krissinel, E.; Henrick, K. Inference of Macromolecular Assemblies from Crystalline State. *J. Mol. Biol.* **2007**, *372*, 774–797, doi:10.1016/j.jmb.2007.05.022.

2. Sievers, F.; Higgins, D.G. Clustal omega, accurate alignment of very large numbers of sequences. *Methods Mol. Biol.* **2014**, *1079*, 105–116, doi:10.1007/978-1-62703-646-7_6.
3. Heo, L.; Lee, H.; Seok, C. GalaxyRefineComplex: Refinement of protein-protein complex model structures driven by interface repacking. *Sci. Rep.* **2016**, *6*, 1–10, doi:10.1038/srep32153.
4. Strelkov, S. V; Schumacher, J.; Burkhard, P.; Aebi, U.; Herrmann, H.; Mu, M.E. Crystal Structure of the Human Lamin A Coil 2B Dimer : Implications for the Head-to-tail Association of Nuclear Lamins. *J. Mol. Biol.* **2004**, *343*, 1067–1080, doi:10.1016/j.jmb.2004.08.093.
5. Strelkov, S. V.; Burkhard, P. Analysis of α-Helical Coiled Coils with the Program TWISTER Reveals a Structural Mechanism for Stutter Compensation. *J. Struct. Biol.* **2002**, *137*, 54–64, doi:10.1006/jsbi.2002.4454.
6. Ahn, J.; Jo, I.; Kang, S. mi; Hong, S.; Kim, S.; Jeong, S.; Kim, Y.H.; Park, B.J.; Ha, N.C. Structural basis for lamin assembly at the molecular level. *Nat. Commun.* **2019**, *10*, 3757, doi:10.1038/s41467-019-11684-x.
7. Fiala, J.; Kukačka, Z.; Novák, P. Influence of cross-linker polarity on selectivity towards lysine side chains. *J. Proteomics* **2020**, *218*, doi:10.1016/j.jprot.2020.103716.

© 2020 by the authors. Submitted for possible open access publication under the terms and conditions of the Creative Commons Attribution (CC BY) license (http://creativecommons.org/licenses/by/4.0/).
